# The distribution and mitochondrial genotype of the hydroid *Aglaophenia latecarinata* is correlated with its pelagic *Sargassum* substrate type in the tropical and subtropical western Atlantic Ocean

**DOI:** 10.7717/peerj.7814

**Published:** 2019-10-18

**Authors:** Annette F. Govindarajan, Laura Cooney, Kerry Whittaker, Dana Bloch, Rachel M. Burdorf, Shalagh Canning, Caroline Carter, Shannon M. Cellan, Fredrik A.A. Eriksson, Hannah Freyer, Grayson Huston, Sabrina Hutchinson, Kathleen McKeegan, Megha Malpani, Alex Merkle-Raymond, Kendra Ouellette, Robin Petersen-Rockney, Maggie Schultz, Amy N.S. Siuda

**Affiliations:** 1 Woods Hole Oceanographic Institution, Woods Hole, MA, USA; 2 Sea Education Association, Woods Hole, MA, USA; 3 Marine Science Discipline, Eckerd College, St. Petersburg, FL, USA

**Keywords:** Hydrozoa, Sargassum, Hydroid, Sargasso Sea, Epiphytes, Aglaophenia

## Abstract

The pelagic brown macroalga *Sargassum* supports rich biological communities in the tropical and subtropical Atlantic region, including a variety of epiphytic invertebrates that grow on the *Sargassum* itself. The thecate hydroid *Aglaophenia latecarinata* is commonly found growing on some, but not all, *Sargassum* forms. In this study, we examined the relationship between *A. latecarinata* and its pelagic *Sargassum* substrate across a broad geographic area over the course of 4 years (2015–2018). The distribution of the most common *Sargassum* forms that we observed (*Sargassum fluitans III* and *S. natans VIII*) was consistent with the existence of distinct source regions for each. We found that *A. latecarinata* hydroids were abundant on both *S. natans VIII* and *S. fluitans III*, and also noted a rare observation of *A. latecarinata* on *S. natans I*. For the hydroids on *S. natans VIII* and *S. fluitans III*, hydroid mitochondrial genotype was strongly correlated with the *Sargassum* substrate form. We found significant population genetic structure in the hydroids, which was also consistent with the distributional patterns of the *Sargassum* forms. These results suggest that hydroid settlement on the *Sargassum* occurs in type-specific *Sargassum* source regions. Hydroid species identification is challenging and cryptic speciation is common in the Aglaopheniidae. Therefore, to confirm our identification of *A. latecarinata*, we conducted a phylogenetic analysis that showed that while the genus *Aglaophenia* was not monophyletic, all *A. latecarinata* haplotypes associated with pelagic *Sargassum* belonged to the same clade and were likely the same species as previously published sequences from Florida, Central America, and one location in Brazil (São Sebastião). A nominal *A. latecarinata* sequence from a second Brazilian location (Alagoas) likely belongs to a different species.

## Introduction

*Sargassum*, a common brown macroalgae, is distributed globally from temperate to tropical ocean waters. Of the more than 350 recognized species (Guiry & Guiry, 2018), *Sargassum natans* and *S. fluitans* are uniquely holopelagic (Butler et al., 1983; Stoner, 1983). These two species have several distinct forms that differ in their bladder and blade characteristics: *S. natans* is comprised of forms *I*, *II*, *VIII*, and *XI*, and *S. fluitans* is comprised of forms *III* and *IV* (Parr, 1939; Schell, Goodwin & Siuda, 2015). *Sargassum natans* and *S. fluitans* lack holdfasts and reproductive structures (Parr, 1939), and new individuals derive from the fragmentation of existing individuals adrift at the sea surface. Pelagic *Sargassum* is ecologically important as an oasis of life on the oligotrophic open ocean. Individual clumps, 10 s of centimeters in each dimension, host abundant epiflora and epifauna that serve as the base of a complex food web similar to those found in benthic habitats (Butler et al., 1983; Coston-Clements et al., 1991). Mats of aggregated clumps measuring 10 s of meters across additionally provide foraging or nursery habitat for fish (Wells & Rooker, 2004), turtles (Witherington, Hirama & Hardy, 2012), and seabirds (Moser & Lee, 2012).

**Figure 1:**
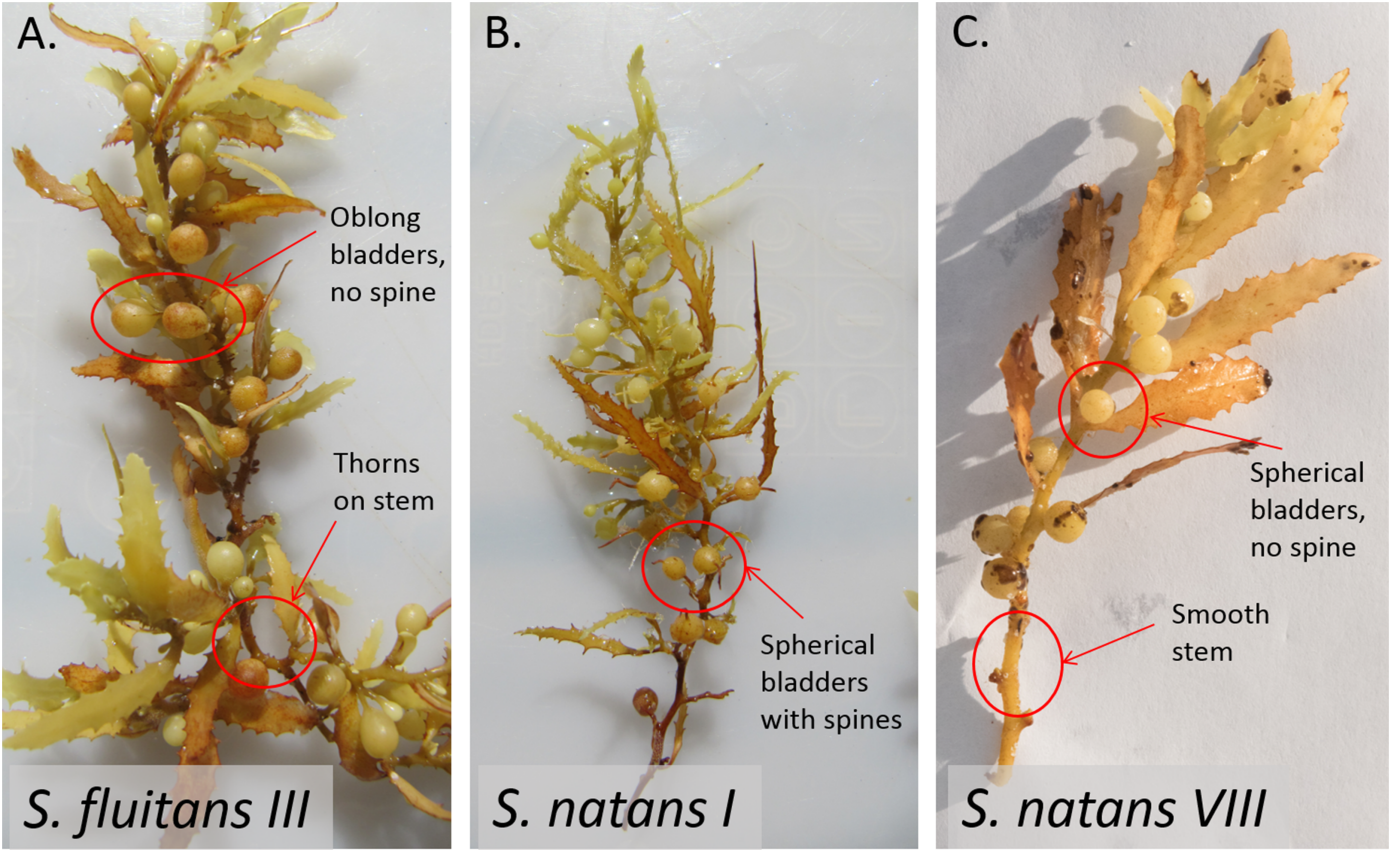
Morphological differences between Sargassum forms as described in Parr (1939) and Schell, Goodwin & Siuda (2015). *S. fluitans* have thorny stems whereas *S. natans* have smooth stems. Bladder and blade attributes differ widely among forms. (A) For *S. fluitans* III, thorns on stem are present, blades are wide, bladders are devoid of spines, and bladders are oblong. (B) For *S. natans I*, stem is smooth, blades are narrow, spines are present on bladders, and bladders are spherical (C) For *S. natans VIII* (photo credit: Janet Bering), stem is smooth, blades are wide, bladders are devoid of spines, and bladders are spherical.

Historically, pelagic *Sargassum* spp. was abundant in the Sargasso Sea and Gulf of Mexico and less abundant or absent in the Caribbean Sea (reviewed in Butler et al., 1983). Since 2011, coastlines on both sides of the tropical Atlantic, including the Caribbean Sea, have experienced three discrete and unprecedented inundations of pelagic *Sargassum*, each lasting many months (Schell, Goodwin & Siuda, 2015; Wang et al., 2019). The most recent event, during 2018, was the most extreme to date (Langin, 2018). Backtracking of landings using archived surface current model data (Franks, Johnson & Ko, 2016), satellite observations (Wang & Hu, 2017; Wang et al., 2019), and biophysical modeling (Brooks et al., 2018) indicate that these recent inundation events originated in the equatorial Atlantic, a new source region for pelagic *Sargassum*. In situ observations revealed that the inundating *Sargassum* was dominated by a previously rare form (*S. natans VIII*) that is morphologically distinct from the two common forms (*S. natans I* and *S. fluitans III*) of pelagic *Sargassum* observed in the Sargasso Sea (Schell, Goodwin & Siuda, 2015; Fig. 1).

**Figure 2:**
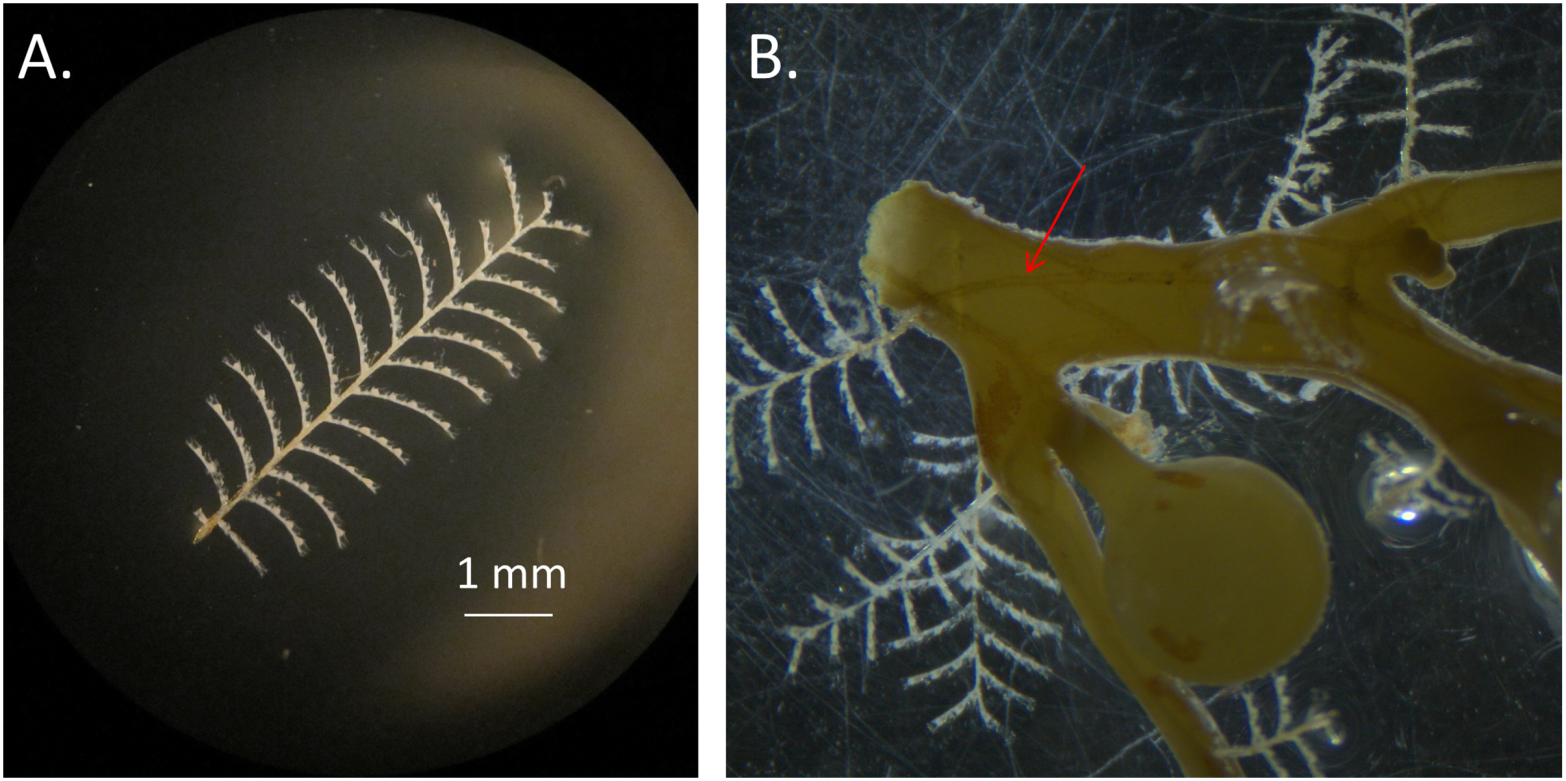
*Aglaophenia latecarinata* hydroids. (A) Isolated *Aglaophenia latecarinata* specimen. (B) A colony of the epiphytic hydroid species, *Aglaophenia latecarinata*, attached to *Sargassum* stem. Arrow points to stolon connecting genetically identical units of a single colony.

*Aglaophenia latecarinata* (Allman, 1877) is a thecate hydroid that is commonly found on pelagic *Sargassum* (Calder, 1993; Calder, 1997; Cunha & Jacobucci, 2010; Fig. 2). In the Sargasso Sea, *A. latecarinata* is a dominant hydrozoan on *S. fluitans III* while it is rare or absent on *S. natans I* (Ryland, 1974; Niermann, 1986). Weis (1968) and Calder (1995) also report the absence of *A. latecarinata* on *S. natans*, though they do not report the type of *S. natans* they examined. *A. latecarinata* has been observed to be abundant, however, on *S. natans VIII* (Burkenroad in Parr, 1939). *A. latecarinata* is found on a variety of substrates in other parts of its range (Oliveira & Marques, 2007; Moura et al., 2018). *Aglaophenia latecarinata* forms feather-like colonies of polyps that can reach up to 10 mm in height (Calder, 1997). Within a colony, polyp fronds are connected via a stolon along the *Sargassum* substrate (Fig. 2). As the species lacks a medusa stage, the hydroids release planula larvae which settle onto nearby substrates and develop into new hydroids (Calder, 1997). For *A. latecarinata* on *Sargassum* substrates floating over deep ocean regions, planulae likely originate from the same or nearby *Sargassum*. Additionally, asexual fragments or propagules from the same or nearby *Sargassum* may also generate new hydroid colonies (Pati & Belmonte, 2018).

Species identification based on morphology in aglaopheniid hydroids is exceptionally challenging (Svoboda & Cornelius, 1991). Recent genetic analyses suggest abundant cryptic speciation in the family and that *A. latecarinata* falls outside the main *Aglaophenia* clade (Postaire et al., 2016; Moura et al., 2018). The geographic range of *A. latecarinata* includes the northwestern Atlantic Ocean including the Sargasso Sea, the Gulf of Mexico, the southwestern Atlantic Ocean, and the western Pacific Ocean (Calder, 1997); however, *A. latecarinata* from the Sargasso Sea has not been included in any of the molecular analyses to date.

Here, we examined the relationship between *A. latecarinata* and its most common pelagic *Sargassum* substrates. We sought to determine whether we could observe population genetic variation as detected by the 16S gene over a vast geographic area in the tropical and subtropical western Atlantic Ocean, and if any observed variation could be associated with region and substrate type. *Aglaophenia latecarinata* was collected from both *S. natans VIII* and *S. fluitans III* from the tropical and subtropical western Atlantic between 2015 and 2018. We report the first observations of abundant *A. latecarinata* on *S. natans VIII* since the 1930s (Burkenroad in Parr, 1939) as well as a single observation of *A. latecarinata* on *S. natans I*. We sequenced the 16S gene from the hydroids on *S. natans VIII* and *S. fluitans III* and found that haplotypes were strongly associated with their *Sargassum* substrate type. We suggest that this finding could reflect hydroid colonization at different *Sargassum* source regions. We also show that in a family-level phylogenetic analysis of 16S sequences, *A. latecarinata* falls outside the main *Aglaophenia* clade and that wide-ranging pelagic *Sargassum-*associated *A. latecarinata* is the same species as individuals collected in previous studies from Florida, Central America, and Sao Sebastiao, Brazil.

## Materials and Methods

### Sampling

**Figure 3:**
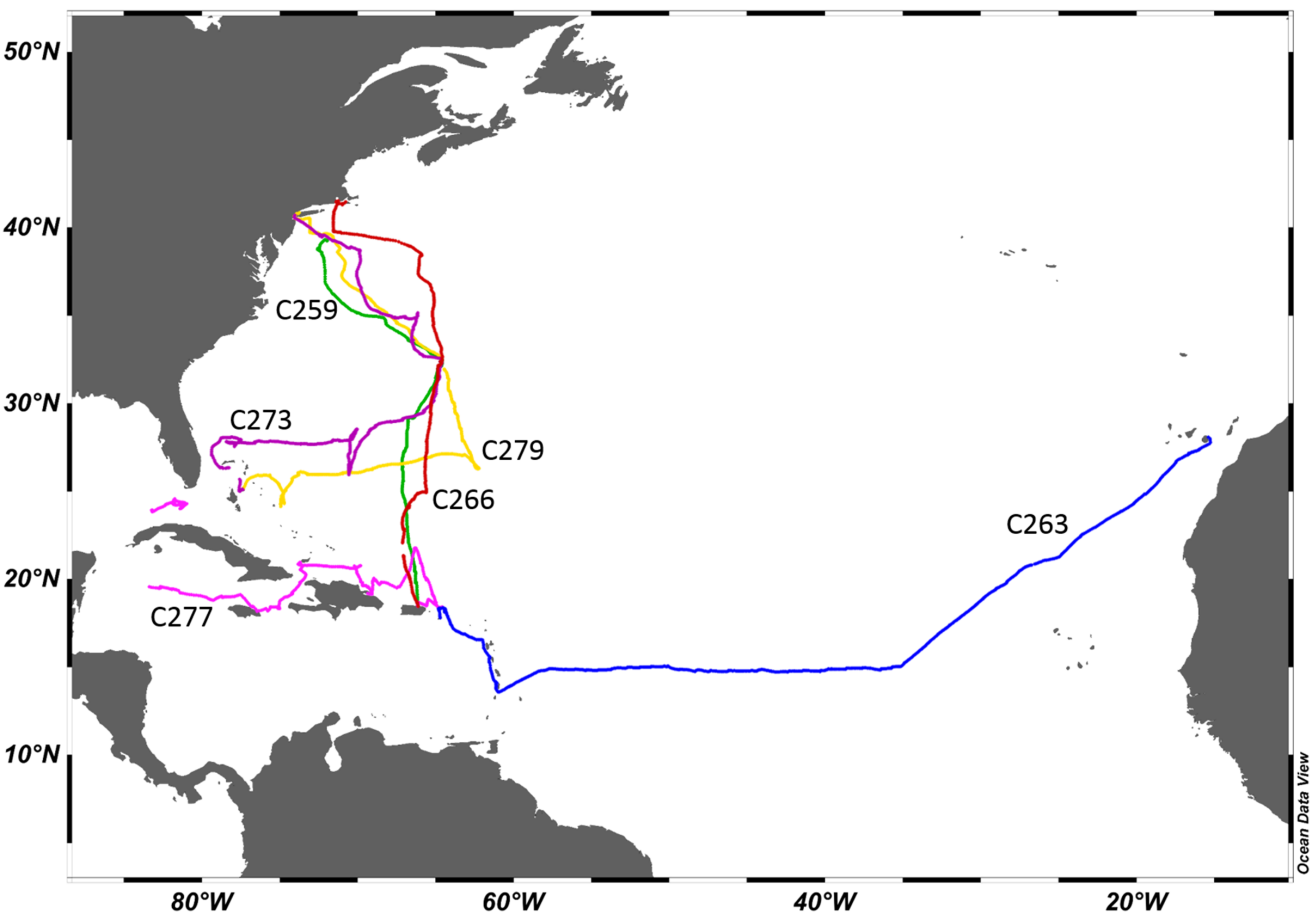
Cruise tracks. Each cruise is represented by a different color. Cruise labels are Sea Education Association cruise numbers. C259 and C263 took place in 2015, C266 took place in 2016, C273 took place in 2017, and C277 and C279 took place in 2018.

*Aglaophenia latecarinata* samples were collected aboard the SSV Corwith Cramer between 2015 and 2018 during Sea Education Association cruises from the Canary Islands to the Caribbean (2015), from San Juan, Puerto Rico to New York, New York (2015) or Woods Hole, Massachusetts (2016) and from Nassau, Bahamas to New York, New York (2017 and 2018) via Bermuda, or from San Juan, Puerto Rico to Key West, Florida (2018) and the cruise tracks were mapped using Ocean Data View 5.1.7 (Schlitzer, 2018) (Fig. 3). Cruise plans were filed with the US State Department, who obtained the required collection permits. No permits were required for sampling in international and US waters under federal jurisdiction. The cruise and permit numbers for the samples collected in this study are as follows: C-259 US State Department Cruise F2014-092, no permits necessary; C-263, US State Department Cruise F2015-044, no permits necessary; C-266, US State Department Cruise F2015-083, no permits necessary; C-273, US State Department Cruise F2016-084, Bermuda permit number SP170104, Bahamas MAMR/FIS/13; C-277, US State Department Cruise F2017-067, Haiti permit number SEMANAH/P-Nav/590, Dominican Republic permit (“Official Letter”) number 26940; and C279, US State Department Cruise F2017-112, Bermuda permit number SP171103 and Bahamas permit number MAMR/FIS/13.

During each cruise, instruments mounted in line with a clean seawater flow-through system (intake at ~3 m) continuously measured temperature and salinity (Sea-Bird Electronics SBE 45 MicroTSG), as well as relative chlorophyll-*a* fluorescence (Turner Designs Model 10-AU in vivo chlorophyll-*a* fluorometer). *Sargassum* specimens were primarily collected opportunistically from dip nets that targeted distinct clumps. Less frequently, *Sargassum* clumps were collected from a neuston net (1 × 0.5 m) with 335 μm mesh towed alongside the ship at two knots for 30 min. We aimed to collect a maximum of 10–12 clumps of each of *Sargassum* form at every station. Each *Sargassum* clump was photographed and identified morphologically using Parr (1939) and Schell, Goodwin & Siuda (2015) (Fig. 1). When present, multiple *A. latecarinata* polyps from one individual hydroid (all connected by a visible stolon, Fig. 2) were plucked from each *Sargassum* clump using sterilized forceps, preserved in 95% ethanol as a single sample, and stored at room temperature. Vouchers of each *Sargassum* sample, including the representative hydroid and epibiont community, were preserved in 95% ethanol. Four representative vouchers that include *A. latecarinata* polyps attached to *S. natans VIII* and *S. fluitans III* substrates were submitted to the Smithsonian Museum of Natural History (USNM catalog numbers 1578893–1578896).

### Sequencing and population analyses

For molecular analysis, we sequenced hydroids found on *S. fluitans III* and *S. natans VIII*. The hydroid colony on *S. natans I* was not included in our analysis given that it was a single observation. Two to three polyps from each hydroid individual were removed from ethanol, rinsed in deionized water, and diced using a sterilized razor blade. Genomic DNA (gDNA) of each hydroid sample was extracted using a Qiagen DNeasy Blood & Tissue Kit (Qiagen, Germantown, MD, USA) following the manufacturer’s protocol, and the final product was eluted twice using 100 µL of AE buffer. A segment of mitochondrial 16S rDNA was amplified using hydrozoan-specific primers: HYD1: 5′-TCG ACT GTT TAC CAA AAA CAT AGC-3′ and HYD2: 5′-ACG GAA TGA ACT CAA ATC ATG TAA G-3′ (Cunningham & Buss, 1993). PCR amplification consisted of an initial temperature of 95 °C for 3 min followed by 35 cycles at 95 °C for 30 s, 45 °C for 30 s and 68 °C for 60 s, and a final extension at 68 °C for 5 min. PCR products were visualized on a 1.5% agarose gel stained with SYBR_®_ Safe (Invitrogen, USA). PCR products were purified using QIAquick PCR Purification kits (Qiagen, Germantown, MD, USA) following the manufacturer’s protocol except that the final elution step was modified to yield 30 µL total volume. Purified amplicons were quantified using a Nanodrop ND-1000 spectrophotometer (Thermo Fisher Scientific, Waltham, MA, USA) and sent to MWG Eurofins Operon (Huntsville, AL) or the DNA Analysis Facility at Yale University (New Haven, CT) for bidirectional Sanger sequencing on an ABI 3730XL capillary sequencer. Sequences were submitted to GenBank (accession numbers MK863834–MK863972).

Geneious versions 9.0.5 and 11.0.5 were used to assemble and curate chromatographs (Biomatters Ltd., Auckland, New Zealand). An alignment was generated with CLUSTALW (Larkin et al., 2007) using the default settings in Geneious. The alignment was trimmed at both ends to remove low-quality sequences. A haplotype network was constructed using TCS version 1.2.1 (Clement, Posada & Crandall, 2000) with gaps treated as a fifth character state.

**Figure 4:**
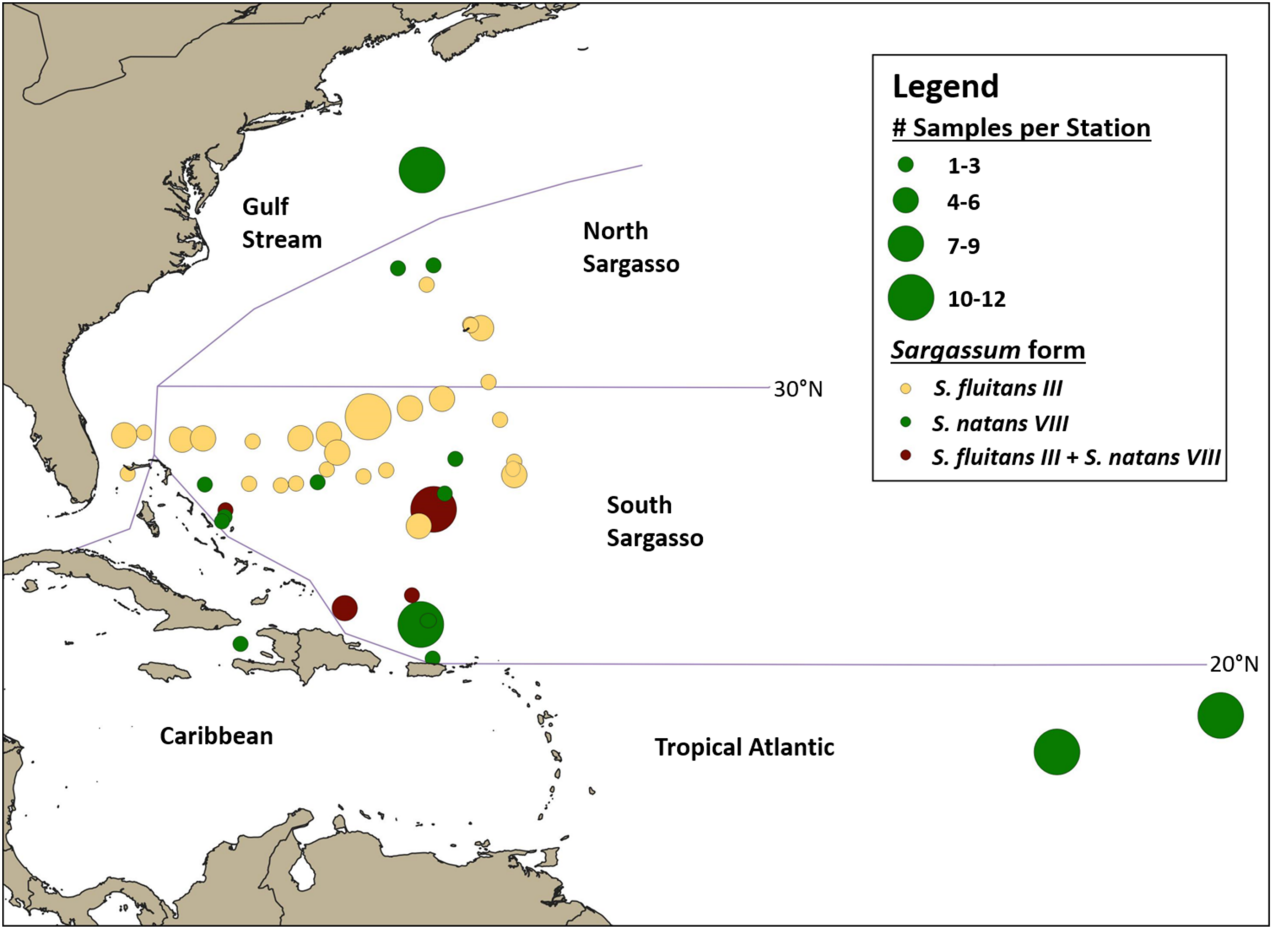
Geographic distribution of hydroid samples by *Sargassum* form. Lines indicate boundaries between oceanographic regions (Gulf Stream, North Sargasso Sea, South Sargasso Sea, Tropical Atlantic, and Caribbean) as described in the text. Each circle represents a single station and circle size corresponds to the number of *Sargassum* samples collected at each station. Yellow indicates stations where only *S. fluitans III* was collected, green indicates stations where only *S. natans VIII* was collected, and maroon indicates stations where both *S. fluitans III* and *S. natans VIII* were collected.

Samples were categorized into broad oceanographic regions using QGIS (QGIS Development Team (2018) (Fig. 4). Samples collected south of 30°N (the approximate location of the subtropical convergence zone; Ullman, Cornillon & Shan, 2007) and north of the Greater Antilles were categorized as from the South Sargasso Sea. Samples collected north of 30°N and south of the Gulf Stream were categorized as from the North Sargasso Sea. Samples collected within the Gulf Stream were categorized as such. Samples collected south of 20°N in or out of the Caribbean were categorized as from the Caribbean or Tropical Atlantic, respectively. An analysis of molecular variance was performed and pairwise *F*_ST_’s calculated with Arlequin ver. 3.5.2 (Excoffier & Lischer, 2010) to test for geographic structure between these regions.

### Phylogenetic analysis

To confirm *A. latecarinata* species identification, we downloaded representative 16S sequences for *A. latecarinata* and other aglaopheniid species from Genbank. Using ClustalW, we constructed an alignment with the Genbank sequences and one representative of each of our haplotypes from our short alignment. The ends of the alignment were trimmed and regions that contained gaps that could not be aligned with confidence were removed. Maximum likelihood and Bayesian analyses on this alignment were conducted using PAUP* (Swofford, 2003) and MrBayes (Huelsenbeck & Ronquist, 2001) through the Geneious interface. The best-fit model for these analyses was selected using Akaike Information Criterion using ModelTest (Posada & Crandall, 1998). In the maximum likelihood analysis, a phylogeny was constructed using the selected model and support for the nodes was determined with a bootstrap analysis with 1,000 replicates. The Bayesian analysis was run with a chain length of 1,100,000, a subsampling frequency of 1,000, a burn-in of 100,000, four heated chains, and a heated chain temperature of 0.2.

## Results

### Sampling

A total of 140 *A. latecarinata* colonies were collected at 47 stations across the Tropical Atlantic, Caribbean, South Sargasso Sea, North Sargasso Sea, and Gulf Stream regions (Fig. 4; Table S1) and sequenced. The distribution of *A. latecarinata* samples across two *Sargassum* forms was nearly even, with 68 colonies collected from *S. natans VIII* and 71 colonies collected from *S. fluitans III*. Only one *A. latecarinata* colony was observed on *S. natans I* (from the North Sargasso Sea in 2015) despite frequent observations of *S. natans I* throughout multiple cruises. The temporal and geographic distribution of *Sargassum* forms represented in the dataset was quite variable due to episodic *S. natans VIII* inundations from the equatorial Atlantic and predetermined cruise tracks (Table 1). However, because dip net sampling was selective, sample density is not necessarily representative of actual *Sargassum* density. For a representative view of *Sargassum* distribution and relative abundance, refer to Schell, Goodwin & Siuda (2015). In 2015, 20 of 21 *A. latecarinata* samples were collected from *S. natans VIII* at stations in the Tropical Atlantic. Of the 40 samples collected in 2016, a majority were again collected from *S. natans VIII*, and some from stations in the South Sargasso Sea (*n* = 20) and northern Gulf Stream (*n* = 12). In contrast, most (43 out of 46) samples in 2017 were collected from *S. fluitans III* at stations in the southern Gulf Stream (*n* = 5), South Sargasso Sea (*n* = 36) and North Sargasso Sea (*n* = 2). In 2018, 11 samples were collected from *S. natans VIII* and 14 samples were collected from *S. fluitans III* in the South Sargasso Sea, while six samples were collected from *S. fluitans III* in the North Sargasso Sea. The number of *Sargassum* clumps collected per station ranged from 1 to 12; only at four of 47 stations (C266-005, C266-011, C277-020, C279-003) were we able to concurrently collect *A. latecarinata* samples from *S. natans VIII* and *S. fluitans III*. These stations were all located in the South Sargasso Sea.

**Table 1:** Sample summary. Number of hydroids samples collected from each *Sargassum* substrate by geographic region.

	2015		2016		2017		2018		Total	
	Sn8	Sf3	Sn8	Sf3	Sn8	Sf3	Sn8	Sf3	Sn8	Sf3
South Sargasso	1	0	20	8	0	36	11	14	32	58
North Sargasso	0	0	0	0	3	2	0	6	3	8
Tropical Atlantic	20	0	0	0	0	0	0	0	20	0
Gulf stream	0	0	12	0	0	5	0	0	12	5
Caribbean	0	0	0	0	0	0	1	0	1	0
Total	21	0	32	8	3	43	12	20	68	71

### Sequence variation

**Figure 5:**
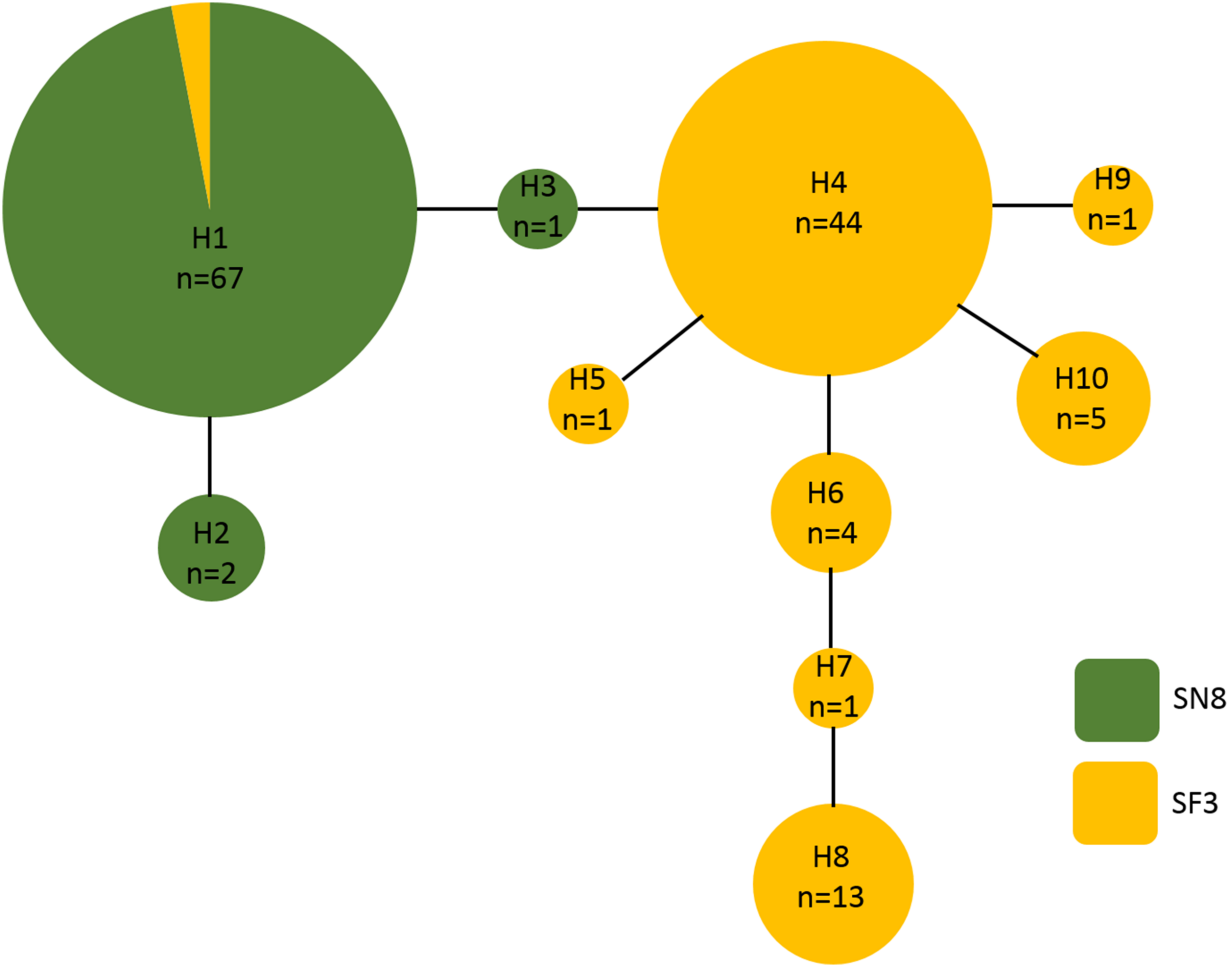
Haplotype network of *Aglaophenia latecarinata* sequences. Circle size reflects the number of individuals possessing a given haplotype (*n*). Yellow indicates hydroids found on *S. fluitans III* and green indicates hydroids found on *S. natans VIII*.

A 500 base pair alignment was obtained after trimming the sequence ends to remove low-quality regions. The alignment had eight variable positions, four of which were indels, that comprised 10 unique haplotypes. Haplotype frequency and diversity were strongly associated with algal substrate, with only one haplotype (haplotype 1) growing on both *S. natans VIII* and *S. fluitans III* (Fig. 5). Of the 68 *A. latecarinata* colonies found on *S. natans* VIII, 65 were haplotype 1, two were haplotype 2, and one was haplotype 3 (Fig. 5). Of the 71 *A. latecarinata* colonies found on *S. fluitans III*, two were haplotype 1, 44 were haplotype 4, one was haplotype 5, four were haplotype 6, one was haplotype 7, 13 were haplotype 8, one was haplotype 9, and five were haplotype 10 (Fig. 5). There were only two colonies collected from *S. fluitans III* that had an “*S. natans VIII*” haplotype (haplotype 1; these were 2016_SF3_41 from the South Sargasso Sea and 2017_SF3_65 from the Gulf Stream, Table S1). No colonies on *S. natans VIII* possessed an “*S. fluitans III*” haplotype. To confirm that our results were consistent over a longer sequence length, we constructed an alternative alignment with a subset of sequences that had expanded high-quality reads (590 base pairs for 99 sequences). The resulting haplotype analysis was consistent with the one based on the shorter, but more inclusive, alignment (Fig. S1).

We found significant geographic structure between regions (Table 2). Pairwise *F*_ST_ values ranged from 0.02030 between the South and North Sargasso Seas, to 0.62631 between the North Sargasso Sea and the Tropical Atlantic (Table 3). All comparisons were significant (*p* < 0.05) except for between the South and North Sargasso Seas.

**Table 2:** Analysis of molecular variance (AMOVA) results.

Source of variation	d*f*	Sum of squares	Variance components	Percentage of variation
Among populations	3	6.171	0.07200 Va	19.77
Within populations	135	39.433	0.29210 Vb	80.23
Total	138	45.604	0.36410	
Fixation index	0.19774			

**Table 3:** Population pairwise *F*_ST_’s (below the diagonal) and *p*-values (above the diagonal).

	TA	SS	NS	GS
TA	–	0.00000 ± 0.0000	0.00000 ± 0.0000	0.01802 ± 0.0182
SS	0.29003	–	0.22523 ± 0.0389	0.00901 ± 0.0091
NS	0.62631	0.02030	–	0.00901 ± 0.0091
GS	0.12084	0.10557	0.24759	–

**Note:**

TA, Tropical Atlantic; SS, South Sargasso Sea; NS, North Sargasso Sea; GS, Gulf Stream.

### Phylogenetic placement

**Figure 6:**
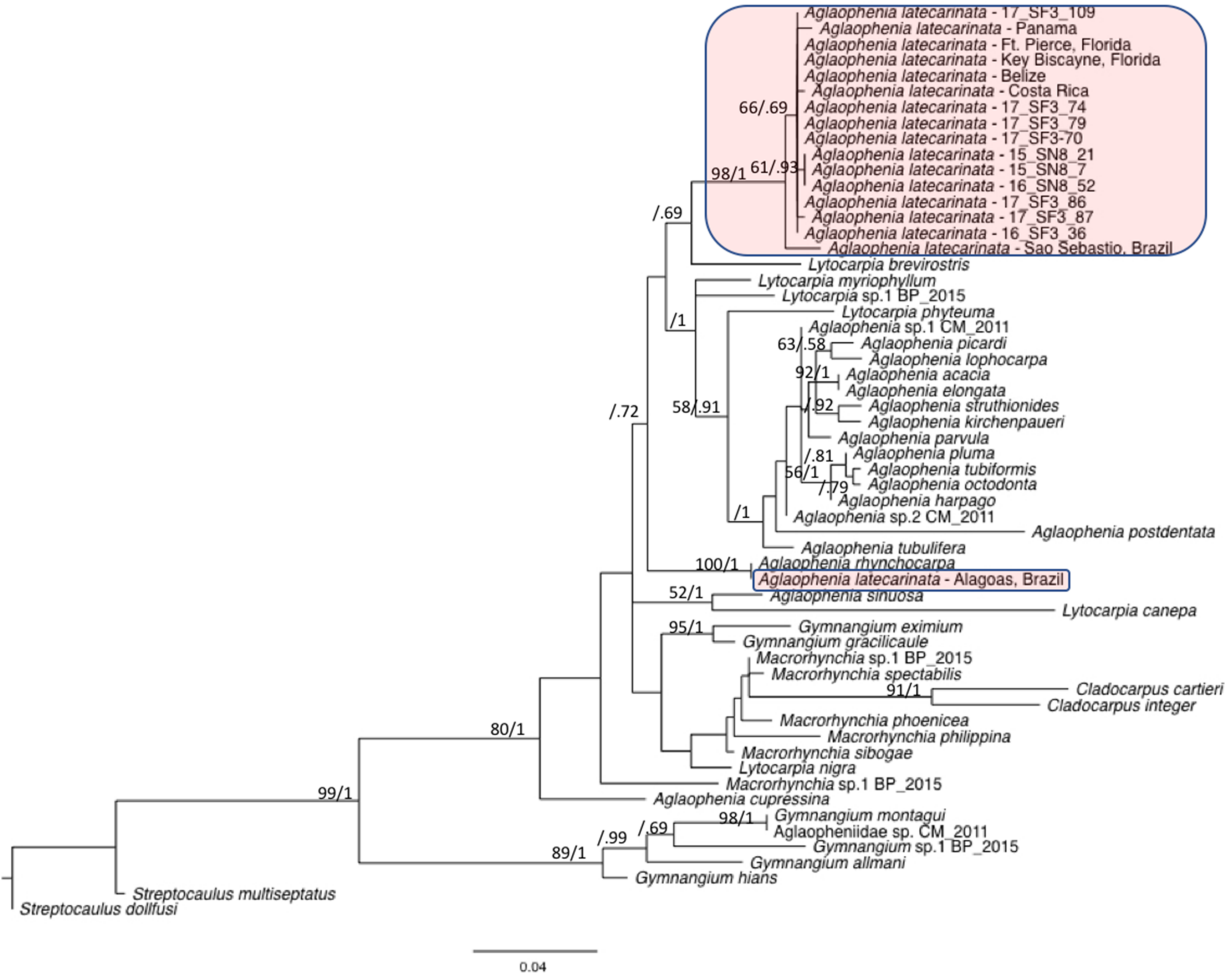
Maximum likelihood phylogeny of the Aglaopheniidae. Support for nodes is indicated by ML bootstrap values before the slash (only those above 50 are shown) and Bayesian posterior probabilities after the slash (only those above 0.5 are shown). *A. latecarinata* sequences are highlighted in red.

We combined representative sequences of each of our *A. latecarinata* haplotypes with 16S sequences from aglaopheniid species on Genbank (Table 4). In instances when multiple sequences for a given species were present, we selected one sequence to represent that species, with the exception of *A. latecarinata*, for which we used all available sequences. The GenBank *A. latecarinata* sequences originated from two locations in the southern hemisphere (São Sebastião and Alagoas, Brazil), and five locations in the northern hemisphere ranging from Panama to Fort Pierce, Florida. The Florida sequence, which interestingly was the only specimen noted to originate from a hydroid colony on *Sargassum*, although the species of *Sargassum* was not given; (Moura et al., 2018), matched one of our haplotypes. The rest of the Genbank *A. latecarinata* sequences were unique. Our trimmed alignment was 378 base pairs. Our maximum likelihood and Bayesian phylogenetic analyses showed that the northern hemisphere sequences (including ours) formed a weakly supported clade (bootstrap and Bayesian posterior probability values were 66 and 0.69, respectively; Fig. 6). This clade, plus the São Sebastião sequence, formed a strongly supported clade (bootstrap and Bayesian posterior probability values were 98 and 1, respectively; Fig. 6). The *A. latecarinata* sequence from Alagoas, Brazil, fell outside of the main *A. latecarinata* clade and clustered strongly with *A. rhynchocarpa* (ML bootstrap and Bayesian posterior probability values were 100 and 1, respectively; Fig. 6). The genus *Aglaophenia* was not monophyletic although support for the arrangement of the nodes was generally weak—often less than 50 in the ML bootstrap analysis, but occasionally higher in the Bayesian analysis (Fig. 6).

**Table 4:** Aglaopheniid sequences from GenBank used in the phylogenetic analysis.

Genus	Species	Reference	GenBank accession#	Location *(A. latecarinata)*
*Aglaophenia*	*latecarinata*	Moura et al. (2018)	MH212420	Carrie Bow Key, Belize
		Moura et al. (2018)	MH212421	Ft. Pierce, FL, USA (on *Sargassum*)
		Moura et al. (2018)	MH212422	Isla Tambor, Panama
		Moura et al. (2018)	MH212423	Isla Uvita, Costa Rica
		Moura et al. (2018)	MH212424	Key Biscayne, FL, USA
		Maronna et al. (2016)	KT266600	Barra do São Miguel, Alagoas, Brazil
		Leclère, Schuchert & Manuel (2007)	DQ855936	Ponta do Baleeiro, São Sebastião, Brazil
	*acacia*	Leclère et al. (2009)	FJ550507	
	*cupressina*	Postaire et al. (2016)	KM587399	
	*elongata*	Leclère et al. (2009)	FJ550508	
	*harpago*	Moura et al. (2012)	JN560129	
	*kirchenpaueri*	Moura et al. (2012)	JN560124	
	*lophocarpa*	Moura et al. (2012)	JN560112	
	*octodonta*	Leclère, Schuchert & Manuel (2007)	DQ855915	
	*parvula*	Moura et al. (2012)	JN560097	
	*picardi*	Moura et al. (2012)	JN560105	
	*pluma*	Moura et al. (2012)	JN560130	
	*postdentata*	Postaire et al. (2016)	KM587408	
	*rhynchocarpa*	Maronna et al. (2016)	KT266601	
	*sinuousa*	Postaire et al. (2016)	KM587411	
	*struthionides*	Maronna et al. (2016)	KT266602	
	*tubiformis*	Leclère, Schuchert & Manuel (2007)	DQ855917	
	*tubulifera*	Moura et al. (2012)	JN560117	
	sp. 1 (CM 2011)	Moura et al. (2012)	JN560094	
	sp. 2 (CM 2011)	Moura et al. (2012)	JN560101	
*Cladocarpus*	*cartieri*	Moura et al. (2012)	JN560085	
	*integer*	Leclère et al. (2009)	FJ550512	
*Gymnangium*	*allmani*	Postaire et al. (2016)	KM587415	
	*eximium*	Postaire et al. (2016)	KM587417	
	*gracilicaule*	Postaire et al. (2016)	KM587438	
	*hians*	Postaire et al. (2016)	KM587446	
	*montagui*	Moura et al. (2012)	JN560075	
	sp. 1 (BP 2015)	Postaire et al. (2016)	KM587460	
*Lytocarpia*	*brevirostris*	Boissin et al. (2018)	MH108512	
	*canepa*	Maronna et al. (2016)	KT266645	
	*myriophyllum*	Moura et al. (2012)	JN560089	
	*nigra*	Postaire et al. (2016)	KM587482	
	*phyteuma*	Postaire et al. (2016)	KM587489	
	sp. 1 (BP 2015)	Postaire et al. (2016)	KM587509	
*Macrorhynchia*	*philippina*	Postaire et al. (2016)	KM587516	
	*phoenicea*	Postaire et al. (2016)	KM587526	
	*sibogae*	Postaire et al. (2016)	KM587537	
	*spectabilis*	Postaire et al. (2016)	KM587539	
	sp. 1 (BP 2015)	Postaire et al. (2016)	KM587538	
	sp. 2 (BP 2015)	Postaire et al. (2016)	KM587510	
*Streptocaulus*	*multiseptatus*	Moura et al. (2012)	JN560080	
	*dollfusi*	Moura et al. (2012)	JN560081	
Aglaopheniidae	sp. (CM 2011)	Moura et al. (2012)	JN560079	

**Note:**

Sampling locations are given for *A. latecarinata* sequences.

## Discussion

Our results add to growing evidence from field and satellite observations that *Sargassum* forms have different source regions and dispersal patterns (Gower & King, 2011; Gower, Young & King, 2013; Schell, Goodwin & Siuda, 2015; Wang & Hu, 2017). The distinct but overlapping geographic ranges of *S. fluitans III* and *S. natans VIII* observed in Schell, Goodwin & Siuda (2015) are also evident in our pattern of opportunistic sample collection. *Sargassum* natans *VIII* was abundant in the Tropical Atlantic and also present in the northern Gulf Stream during some years. *Sargassum* natans *VIII* and *S. fluitans III* co-occurred in the South Sargasso Sea.

Prior to 2011, the primary forms of *Sargassum* in the Sargasso Sea were *S. fluitans III* and *S. natans I* (Schell, Goodwin & Siuda, 2015). Satellite data from this time suggests these forms range from the Caribbean to the Sargasso Sea and likely originate from the Gulf of Mexico (Gower & King, 2011). In contrast, *S. natans VIII* is likely transported from its source in the North Equatorial Recirculation Region via the North Equatorial Current, which splits into the Caribbean Current in the Caribbean Sea and the Antilles Current that runs north of the Greater Antilles (Franks, Johnson & Ko, 2016; Putman et al., 2018; Brooks et al., 2018; Wang et al., 2019). *Sargassum* natans *VIII* from the Caribbean may then be carried into the Gulf of Mexico and on to the Gulf Stream, where it could mix and travel north along with the *S. natans VIII* from the Antilles Current (Wang et al., 2019). Because the boundary between the Antilles Current and South Sargasso Sea is weak, both *S. fluitans III* and *S. natans VIII* are commonly found in this region (Schell, Goodwin & Siuda, 2015).

The seasonality of the blooms and their associated dispersal may also contribute to the maintenance of distinct distributions of each *Sargassum* type and their associated *A. latecarinata* genotypes. Both the Gulf of Mexico and the tropical Atlantic exhibit *Sargassum* blooms in spring (Wang et al., 2019). We suggest that the Gulf of Mexico *Sargassum* (which likely includes *S. fluitans III*) may be exported to the North Atlantic before the tropical Atlantic *Sargassum* (which is likely *S. natans VIII*) arrives there, thus minimizing the potential for interaction of their associated hydroids. Additional sampling of both the hydroids and the *Sargassum* in the Gulf of Mexico and equatorial Atlantic source regions will be necessary to test our proposed mechanism for maintaining distinct *A. latecarinata* populations.

Our frequent observations of *A. latecarinata* on *S. fluitans III* and *S. natans VIII* and single observation of *A. latecarinata* on *S. natans I* are consistent with previous findings. Burkenroad (in Parr, 1939) reported that *A. latecarinata* (reported as *A. minuta*) was the dominant hydroid on both *S. fluitans III* and *S. natans VIII*. Ryland (1974) and Niermann (1986) also did not report *A. latecarinata* on their surveys of epibionts on *S. natans I*. Weis (1968) did not find *A. latecarinata* on *S. natans* and Calder (1995) identified *A. latecarinata* as the dominant hydrozoan on *S. fluitans*, but noted that it was entirely absent from *S. natans*. Neither Weis (1968) nor Calder (1995) specified the type of *S. natans* that they observed, but we suggest that it was likely the *S. natans I* form. Settlement specificity of hydroid species has been observed both within and between other *Sargassum* species (Nishihira, 1965; Nishihira, 1971; Calder, 1995).

The species-specific substrate pattern that we observed could be due to several factors including substrate selection by planula larvae or substrate availability. For example, larvae of the epiphytic hydroid *Coryne uchidae* (Stechow) showed larval settlement preferences when presented with multiple algal substrates including different non-pelagic *Sargassum* species (Nishihira, 1968a). Substrate selection in *C. uchidae* appears to be influenced by chemical cues present in substrate extracts (Nishihira, 1968b; Kato et al., 1975). We are not aware of any similar experiment with *A. latecarinata* larvae. However, *A. latecarinata* hydroids have been successfully transplanted on to *S. natans I* (Burkenroad in Parr, 1939) suggesting that the hydroids can grow on this *Sargassum* form if settlement occurs. If the different *Sargassum* species have distinct source regions as recent research suggests (Schell, Goodwin & Siuda, 2015; Franks, Johnson & Ko, 2016; Wang & Hu, 2017), and larval settlement occurs in these source regions where the *Sargassum* species are not found together, it is possible that substrate availability is primarily responsible for our, and previous, observations. Hydroid colonies could also potentially originate asexually via dislodged fragments attaching to *Sargassum*, and this could lead to species-specific patterns if re-attachment were substrate-specific. More research is needed to determine the degree of substrate specificity as well as the mechanisms driving any specificity, both for pelagic *A. latecarinata* and for aglaopheniids in general.

For the *S. fluitans III* and *S. natans VIII*, which had abundant *A. latecarinata* colonization, we found a striking correlation between hydroid mitochondrial genotype and *Sargassum* species. We also found significant population genetic structure in *A. latecarinata* between North Atlantic regions, which was likely due to the distribution of the *Sargassum* substrates. These findings are consistent with the hypothesis that substrate availability associated with *Sargassum* species source regions is driving *A. latecarinata* colonization patterns. However, the detection of two *S. fluitans III*-derived *A. latecarinata* colonies in haplotype 1, which was the most common haplotype for *S. natans VIII*-derived colonies, points to the potential for limited genetic exchange to take place when the *Sargassum* forms co-occur. We found that *S. fluitans III* and *S. natans VIII* were sometimes found together at the same sampling site in a given year, as was the case for one of the *S. fluitans III*-derived *A. latecarinata* colonies with haplotype 1. While we did not simultaneously collect *S. natans VIII* along with the other *S. fluitans III*-derived *A. latecarinata* colony with haplotype 1, it is possible that it had encountered *S. natans VIII* previously as it drifted through other regions. As such, the dominant population genetic pattern observed in *A. latecarinata* is likely maintained through settlement on substrates that differ in geographic origins.

*Aglaophenia* species typically lack a planktonic medusa stage (Svoboda & Cornelius, 1991) and have been hypothesized to have limited dispersal capabilities, which could lead to population genetic structuring and speciation (Postaire et al., 2016). However, benthic or fixed stages can also disperse via rafting on algal or other substrates (Ronowicz, Kukliński & Mapstone, 2015; Boissin et al., 2018). In contrast to the pattern observed in *A. latecarinata*, the *Sargassum* shrimp *Latreutes fucorum*, which has a long-lived planktonic larval period, exhibited no population genetic structure over the same region (Sehein et al., 2014). This finding could reflect the greater potential of *L. fucorum* to disperse independently of *Sargassum*, and reinforces our hypothesis that substrate availability is an important driver of *A. latecarinata* settlement patterns.

Several studies that utilize 16S sequences suggest cryptic species are common in aglaopheniid taxa. Schuchert (2014) found that in the aglaopheniid genus *Plumularia*, nominal morphologically-defined species showed a high degree of genetic variability indicating possible cryptic speciation. Postaire et al. (2016), employing multiple species delimitation methods, found significant variation in mitochondrial 16S sequences within aglaopheniid morphospecies, which also likely indicates cryptic species. In another study of the family, Moura et al. (2012) found that their 16S *A. latecarinata* sequence from Brazil fell outside of the *Aglaophenia* clade. Our family-level genetic analysis suggests that the different pelagic *Sargassum*-associated hydroid genotypes represent intraspecific variation and not cryptic species. Furthermore, our results show that these *Sargassum*-associated specimens are also likely the same species as those collected from Central America and Florida in Moura et al. (2018) and from São Sebastião in Brazil (Leclère, Schuchert & Manuel, 2007). However, the sequence from Alagaos, Brazil (Maronna et al., 2016) fell well outside the *A. latecarinata* clade and so may represent a cryptic species or a misidentification. At a deeper level, our phylogeny indicated that the genus *Aglaophenia* is polyphyletic, as in previous studies (Moura et al., 2018). Taxonomic studies, coupled with molecular analyses utilizing multiple genetic markers, will be necessary to fully understand aglaopheniid diversity and evolutionary relationships.

## Conclusions

*Aglaophenia* latecarinata hydroids were abundant on *S. natans VIII* and *S. fluitans III*, but rare on *S. natans I*. For the hydroids on *S. natans VIII* and *S. fluitans III*, hydroid mitochondrial genotype was strongly correlated with *Sargassum* substrate form. There was significant population genetic structure in the hydroids, which likely reflects the distribution of their different algal substrates, with *S. natans VIII* likely annually sourced primarily from the equatorial Atlantic and *S. fluitans III* likely annually sourced primarily from the Gulf of Mexico. As cryptic speciation appears to be common in aglaopheniids, we conducted a family-level phylogenetic analysis that showed that the genus *Aglaophenia* was polyphyletic, and that all *A. latecarinata* haplotypes associated with pelagic *Sargassum* belonged to the same clade as published sequences from Florida, Central America, and one location in Brazil (São Sebastião). A nominal *A. latecarinata* sequence from a second Brazilian location (Alagoas) likely belongs to a different species.

## Supplemental Information

10.7717/peerj.7814/supp-1 Supplemental Information 1 Haplotype network based on a longer alignment (590 base pairs) and fewer specimens (99 sequences). Circle size reflects the number of individuals possessing a given haplotype (*n*). Yellow indicates hydroids found on *S. fluitans III* and green indicates hydroids found on *S. natans VIII*.

10.7717/peerj.7814/supp-2 Supplemental Information 2 List of specimens and associated data.

## Additional Information and Declarations

### Field Study Permissions

The following information was supplied relating to field study approvals (i.e., approving body and any reference numbers): Cruise plans were filed with the US State Department, who obtained the required collection permits. No permits were required for sampling in international and US waters under federal jurisdiction.The cruise and permit numbers for the samples collected in this study are as follows: C-259 US State Department Cruise F2014-092, no permits necessary; C-263, US State Department Cruise F2015-044, no permits necessary; C-266, US State Department Cruise F2015-083, no permits necessary; C-273, US State Department Cruise F2016-084, Bermuda permit number SP170104, Bahamas MAMR/FIS/13; C-277, US State Department Cruise F2017-067, Haiti permit number SEMANAH/P-Nav/590, Dominican Republic permit (Official Letter) number 26940; and C279, US State Department Cruise F2017-112, Bermuda permit number SP171103 and Bahamas permit number MAMR/FIS/13.

### Data Availability

The following information was supplied regarding data availability: Sequences are available at GenBank: MK863834–MK863972.
