# Supplementary figures and images for: The distribution and mitochondrial genotype of the hydroid Aglaophenia latecarinata is correlated with its pelagic Sargassum substrate type in the tropical and subtropical western Atlantic Ocean
Source: PeerJ. 2019 Oct 18;7:e7814. doi: 10.7717/peerj.7814 (PMC6802585; doi:10.7717/peerj.7814)

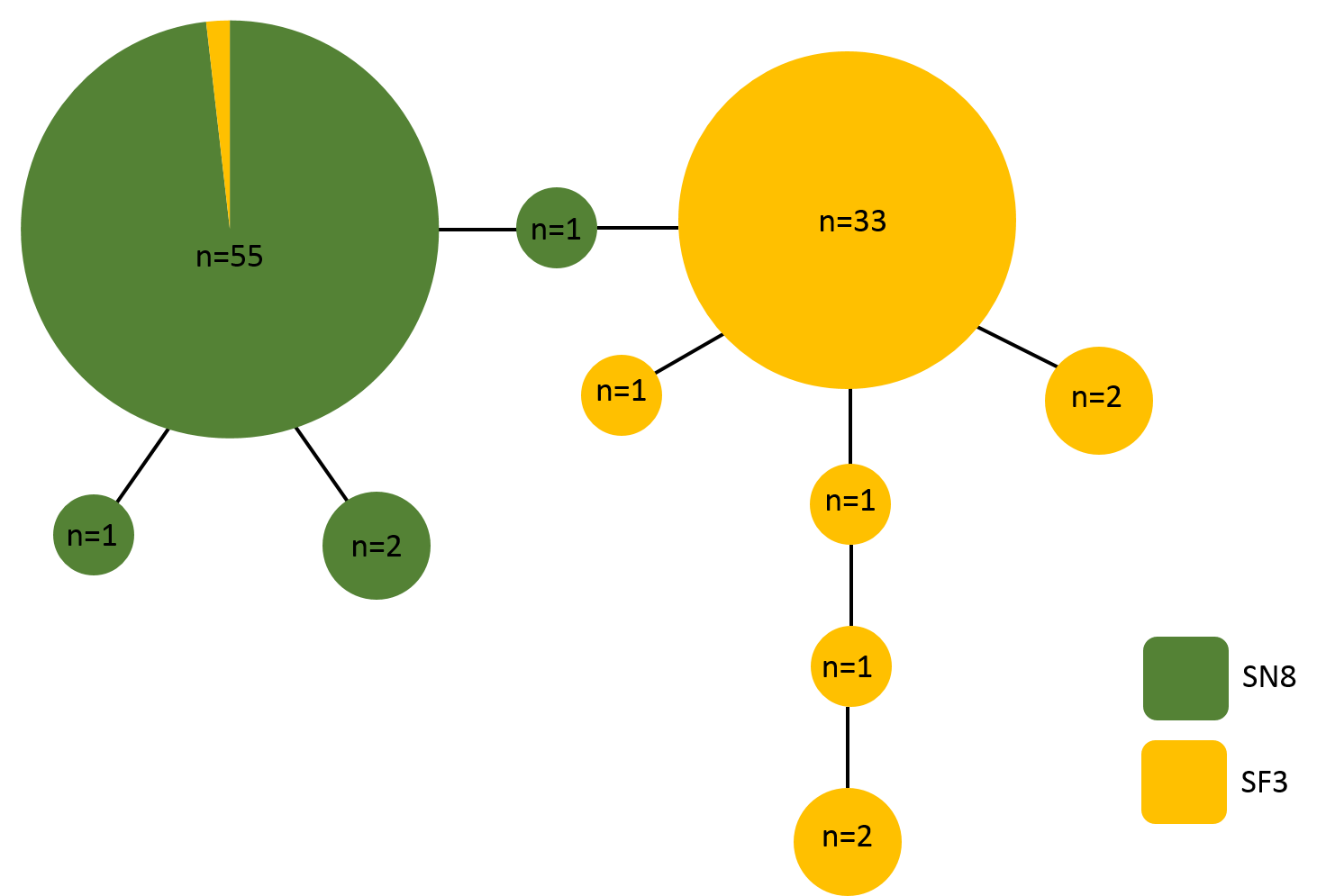

Supplement: Supplemental Information 1 — Circle size reflects the number of individuals possessing a given haplotype (n). Yellow indicates hydroids found on S. fluitans III and green indicates hydroids found on S. natans VIII. [file peerj-07-7814-s001.png]
