# Supplementary material for: The distribution and mitochondrial genotype of the hydroid Aglaophenia latecarinata is correlated with its pelagic Sargassum substrate type in the tropical and subtropical western Atlantic Ocean
Source: PeerJ. 2019 Oct 18;7:e7814. doi: 10.7717/peerj.7814 (PMC6802585; doi:10.7717/peerj.7814)
Supplement: Supplemental Information 2 [file peerj-07-7814-s002.docx]

| **Year** | **Seq ID** | **Latitude** | **Longitude** | **Temp (°C)** | **Salinity (ppt)** | **Fluorescence (RFU)** | **Region** | ***Sargassum* Type** | **NCBI ID** |
| --- | --- | --- | --- | --- | --- | --- | --- | --- | --- |
|  | 2015_SN8_1 | 20.433 | -66.302 | 27.1 | 36.32 | 494.0 | S Sargasso | *S. natans VIII* | MK863834 |
| 2015 | 2015_SN1_2 | 35.170 | -70.028 | 21.3 | 36.62 | 600.7 | N Sargasso | *S. natans I* |  |
|  | 2015_SN8_3 | 16.322 | -33.450 | 26.7 | 36.62 | 712.1 | Tropical Atlantic | *S. natans VIII* | MK863835 |
|  | 2015_SN8_4 |  |  |  |  |  | Tropical Atlantic | *S. natans VIII* | MK863836 |
|  | 2015_SN8_5 |  |  |  |  |  | Tropical Atlantic | *S. natans VIII* | MK863837 |
|  | 2015_SN8_6 |  |  |  |  |  | Tropical Atlantic | *S. natans VIII* | MK863838 |
|  | 2015_SN8_7 |  |  |  |  |  | Tropical Atlantic | *S. natans VIII* | MK863839 |
|  | 2015_SN8_8 |  |  |  |  |  | Tropical Atlantic | *S. natans VIII* | MK863840 |
|  | 2015_SN8_9 |  |  |  |  |  | Tropical Atlantic | *S. natans VIII* | MK863841 |
|  | 2015_SN8_10 |  |  |  |  |  | Tropical Atlantic | *S. natans VIII* | MK863842 |
|  | 2015_SN8_11 |  |  |  |  |  | Tropical Atlantic | *S. natans VIII* | MK863843 |
|  | 2015_SN8_12 |  |  |  |  |  | Tropical Atlantic | *S. natans VIII* | MK863844 |
|  | 2015_SN8_13 | 14.812 | -40.252 | 26.9 | 36.44 | 739.2 | Tropical Atlantic | *S. natans VIII* | MK863845 |
|  | 2015_SN8_14 |  |  |  |  |  | Tropical Atlantic | *S. natans VIII* | MK863846 |
|  | 2015_SN8_15 |  |  |  |  |  | Tropical Atlantic | *S. natans VIII* | MK863847 |
|  | 2015_SN8_16 |  |  |  |  |  | Tropical Atlantic | *S. natans VIII* | MK863848 |
|  | 2015_SN8_17 |  |  |  |  |  | Tropical Atlantic | *S. natans VIII* | MK863849 |
|  | 2015_SN8_18 |  |  |  |  |  | Tropical Atlantic | *S. natans VIII* | MK863850 |
|  | 2015_SN8_19 |  |  |  |  |  | Tropical Atlantic | *S. natans VIII* | MK863851 |
|  | 2015_SN8_20 |  |  |  |  |  | Tropical Atlantic | *S. natans VIII* | MK863852 |
|  | 2015_SN8_21 |  |  |  |  |  | Tropical Atlantic | *S. natans VIII* | MK863853 |
|  | 2016_SN8_22 |  |  |  |  |  | Tropical Atlantic | *S. natans VIII* | MK863854 |
| 2016 | 2016_SN8_23 | 18.682 | -66.162 | 27.2 | 36.398 | 487.0 | S Sargasso | *S. natans VIII* | MK863855 |
|  | 2016_SN8_24 | 20.093 | -66.653 | 27.1 | 36.38 | 514.0 | S Sargasso | *S. natans VIII* | MK863856 |
|  | 2016_SN8_25 |  |  |  |  |  | S Sargasso | *S. natans VIII* | MK863857 |
|  | 2016_SN8_26 |  |  |  |  |  | S Sargasso | *S. natans VIII* | MK863858 |
|  | 2016_SN8_27 |  |  |  |  |  | S Sargasso | *S. natans VIII* | MK863859 |
|  | 2016_SN8_28 |  |  |  |  |  | S Sargasso | *S. natans VIII* | MK863860 |
|  | 2016_SN8_29 |  |  |  |  |  | S Sargasso | *S. natans VIII* | MK863861 |
|  | 2016_SN8_30 |  |  |  |  |  | S Sargasso | *S. natans VIII* | MK863862 |
|  | 2016_SN8_31 |  |  |  |  |  | S Sargasso | *S. natans VIII* | MK863863 |
|  | 2016_SN8_32 |  |  |  |  |  | S Sargasso | *S. natans VIII* | MK863864 |
|  | 2016_SN8_33 |  |  |  |  |  | S Sargasso | *S. natans VIII* | MK863865 |
|  | 2016_SF3_34 | 21.307 | -67.022 | 27.0 | 36.54 | 496.0 | S Sargasso | *S. fluitans III* | MK863866 |
|  | 2016_SF3_35 |  |  |  |  |  | S Sargasso | *S. fluitans III* | MK863867 |
|  | 2016_SF3_36 |  |  |  |  |  | S Sargasso | *S. fluitans III* | MK863868 |
|  | 2016_SN8_37 |  |  |  |  |  | S Sargasso | *S. natans VIII* | MK863869 |
|  | 2016_SF3_38 | 24.105 | -66.705 | 25.6 | 36.16 | 603.0 | S Sargasso | *S. fluitans III* | MK863870 |
|  | 2016_SF3_39 |  |  |  |  |  | S Sargasso | *S. fluitans III* | MK863871 |
|  | 2016_SF3_40 |  |  |  |  |  | S Sargasso | *S. fluitans III* | MK863872 |
|  | 2016_SF3_41 | 24.867 | -66.138 | 25.3 | 36.73 | 565.0 | S Sargasso | *S. fluitans III* | MK863873 |
|  | 2016_SN8_43 |  |  |  |  |  | S Sargasso | *S. natans VIII* | MK863874 |
|  | 2016_SN8_44 |  |  |  |  |  | S Sargasso | *S. natans VIII* | MK863875 |
|  | 2016_SF3_45 |  |  |  |  |  | S Sargasso | *S. fluitans III* | MK863876 |
|  | 2016_SN8_46 |  |  |  |  |  | S Sargasso | *S. natans VIII* | MK863877 |
|  | 2016_SN8_47 |  |  |  |  |  | S Sargasso | *S. natans VIII* | MK863878 |
|  | 2016_SN8_48 |  |  |  |  |  | S Sargasso | *S. natans VIII* | MK863879 |
|  | 2016_SN8_49 |  |  |  |  |  | S Sargasso | *S. natans VIII* | MK863880 |
|  | 2016_SN8_50 |  |  |  |  |  | S Sargasso | *S. natans VIII* | MK863881 |
|  | 2016_SN8_51 | 25.522 | -65.660 | 25.2 | 36.77 | 546.0 | S Sargasso | *S. natans VIII* | MK863882 |
|  | 2016_SN8_52 | 38.940 | -66.615 | 19.6 | 36.07 | 745.6 | Gulf Stream | *S. natans VIII* | MK863883 |
|  | 2016_SN8_53 |  |  |  |  |  | Gulf Stream | *S. natans VIII* | MK863884 |
|  | 2016_SN8_54 |  |  |  |  |  | Gulf Stream | *S. natans VIII* | MK863885 |
|  | 2016_SN8_55 |  |  |  |  |  | Gulf Stream | *S. natans VIII* | MK863886 |
|  | 2016_SN8_56 |  |  |  |  |  | Gulf Stream | *S. natans VIII* | MK863887 |
|  | 2016_SN8_58 |  |  |  |  |  | Gulf Stream | *S. natans VIII* | MK863888 |
|  | 2016_SN8_59 |  |  |  |  |  | Gulf Stream | *S. natans VIII* | MK863889 |
|  | 2016_SN8_60 |  |  |  |  |  | Gulf Stream | *S. natans VIII* | MK863890 |
|  | 2016_SN8_61 |  |  |  |  |  | Gulf Stream | *S. natans VIII* | MK863891 |
|  | 2016_SN8_62 |  |  |  |  |  | Gulf Stream | *S. natans VIII* | MK863892 |
|  | 2016_SN8_63 |  |  |  |  |  | Gulf Stream | *S. natans VIII* | MK863893 |
|  | 2016_SN8_64 |  |  |  |  |  | Gulf Stream | *S. natans VIII* | MK863894 |
| 2017 | 2017_SF3_65 | 26.347 | -78.820 | 25.9 | 36.57 | 555.4 | Gulf Stream | *S. fluitans III* | MK863895 |
|  | 2017_SF3_67 | 27.978 | -78.778 | 25.3 | 36.65 | 671.9 | Gulf Stream | *S. fluitans III* | MK863896 |
|  | 2017_SF3_68 |  |  |  |  |  | Gulf Stream | *S. fluitans III* | MK863897 |
|  | 2017_SF3_69 |  |  |  |  |  | Gulf Stream | *S. fluitans III* | MK863898 |
|  | 2017_SF3_70 | 28.052 | -78.143 | 25.4 | 36.61 | 702.3 | Gulf Stream | *S. fluitans III* | MK863899 |
|  | 2017_SF3_71 | 27.760 | -76.562 | 25.2 | 36.62 | 730.2 | S Sargasso | *S. fluitans III* | MK863900 |
|  | 2017_SF3_72 |  |  |  |  |  | S Sargasso | *S. fluitans III* | MK863901 |
|  | 2017_SF3_73 |  |  |  |  |  | S Sargasso | *S. fluitans III* | MK863902 |
|  | 2017_SF3_74 | 27.808 | -75.692 | 23.6 | 36.88 | 705.8 | S Sargasso | *S. fluitans III* | MK863903 |
|  | 2017_SF3_75 |  |  |  |  |  | S Sargasso | *S. fluitans III* | MK863904 |
|  | 2017_SF3_76 |  |  |  |  |  | S Sargasso | *S. fluitans III* | MK863905 |
|  | 2017_SF3_77 |  |  |  |  |  | S Sargasso | *S. fluitans III* | MK863906 |
|  | 2017_SF3_78 | 27.683 | -73.635 | 23.8 | 36.90 | 622.7 | S Sargasso | *S. fluitans III* | MK863907 |
|  | 2017_SF3_79 | 27.812 | -71.663 | 23.8 | 36.92 | 627.3 | S Sargasso | *S. fluitans III* | MK863908 |
|  | 2017_SF3_80 |  |  |  |  |  | S Sargasso | *S. fluitans III* | MK863909 |
|  | 2017_SF3_81 |  |  |  |  |  | S Sargasso | *S. fluitans III* | MK863910 |
|  | 2017_SF3_82 | 27.812 | -71.520 | 23.7 | 36.92 | 626.6 | S Sargasso | *S. fluitans III* | MK863911 |
|  | 2017_SF3_86 | 27.967 | -70.475 | 23.8 | 36.92 | 625.7 | S Sargasso | *S. fluitans III* | MK863912 |
|  | 2017_SF3_87 |  |  |  |  |  | S Sargasso | *S. fluitans III* | MK863913 |
|  | 2017_SF3_88 |  |  |  |  |  | S Sargasso | *S. fluitans III* | MK863914 |
|  | 2017_SF3_89 |  |  |  |  |  | S Sargasso | *S. fluitans III* | MK863915 |
|  | 2017_SF3_91 | 26.522 | -70.555 | 24.5 | 36.86 | 626.8 | S Sargasso | *S. fluitans III* | MK863916 |
|  | 2017_SF3_92 |  |  |  |  |  | S Sargasso | *S. fluitans III* | MK863917 |
|  | 2017_SF3_93 | 27.222 | -70.125 | 24.1 | 36.92 | 581.1 | S Sargasso | *S. fluitans III* | MK863918 |
|  | 2017_SF3_94 |  |  |  |  |  | S Sargasso | *S. fluitans III* | MK863919 |
|  | 2017_SF3_95 |  |  |  |  |  | S Sargasso | *S. fluitans III* | MK863920 |
|  | 2017_SF3_96 | 28.317 | -69.278 | 23.9 | 36.92 | 814.8 | S Sargasso | *S. fluitans III* | MK863921 |
|  | 2017_SF3_98 | 28.705 | -68.843 | 23.8 | 36.95 | 574.3 | S Sargasso | *S. fluitans III* | MK863922 |
|  | 2017_SF3_99 |  |  |  |  |  | S Sargasso | *S. fluitans III* | MK863923 |
|  | 2017_SF3_100 |  |  |  |  |  | S Sargasso | *S. fluitans III* | MK863924 |
|  | 2017_SF3_101 |  |  |  |  |  | S Sargasso | *S. fluitans III* | MK863925 |
|  | 2017_SF3_102 |  |  |  |  |  | S Sargasso | *S. fluitans III* | MK863926 |
|  | 2017_SF3_103 |  |  |  |  |  | S Sargasso | *S. fluitans III* | MK863927 |
|  | 2017_SF3_104 |  |  |  |  |  | S Sargasso | *S. fluitans III* | MK863928 |
|  | 2017_SF3_105 |  |  |  |  |  | S Sargasso | *S. fluitans III* | MK863929 |
|  | 2017_SF3_106 | 29.055 | -67.105 | 23.6 | 36.95 | 630.6 | S Sargasso | *S. fluitans III* | MK863930 |
|  | 2017_SF3_107 |  |  |  |  |  | S Sargasso | *S. fluitans III* | MK863931 |
|  | 2017_SF3_108 |  |  |  |  |  | S Sargasso | *S. fluitans III* | MK863932 |
|  | 2017_SF3_109 | 29.455 | -65.758 | 23.9 | 36.89 | 582.2 | S Sargasso | *S. fluitans III* | MK863933 |
|  | 2017_SF3_110 |  |  |  |  |  | S Sargasso | *S. fluitans III* | MK863934 |
|  | 2017_SF3_111 |  |  |  |  |  | S Sargasso | *S. fluitans III* | MK863935 |
|  | 2017_SF3_112 | 32.535 | -64.605 | 22.5 | 36.85 | 598.0 | N Sargasso | *S. fluitans III* | MK863936 |
|  | 2017_SF3_113 | 34.183 | -66.410 | 22.2 | 36.73 | 598.3 | N Sargasso | *S. fluitans III* | MK863937 |
|  | 2017_SN8_115 | 34.983 | -66.125 | 21.4 | 36.74 | 595.2 | N Sargasso | *S. natans VIII* | MK863938 |
|  | 2017_SN8_116 | 34.862 | -67.602 | 21.0 | 36.67 | 576.6 | N Sargasso | *S. natans VIII* | MK863939 |
|  | 2017_SN8_117 |  |  |  |  |  | N Sargasso | *S. natans VIII* | MK863940 |
| 2018 | 2018_SN8_119 | 20.772 | -69.812 | 25.50 | 36.97 | 752.90 | S Sargasso | *S. natans VIII* | MK863941 |
|  | 2018_SF3_120 |  |  |  |  |  | S Sargasso | *S. fluitans III* | MK863942 |
|  | 2018_SF3_121 |  |  |  |  |  | S Sargasso | *S. natans VIII* | MK863943 |
|  | 2018_SF3_122 |  |  |  |  |  | S Sargasso | *S. fluitans III* | MK863944 |
|  | 2018_SN8_123 |  |  |  |  |  | S Sargasso | *S. natans VIII* | MK863945 |
|  | 2018_SN8_124 |  |  |  |  |  | S Sargasso | *S. natans VIII* | MK863946 |
|  | 2018_SN8_125 | 19.290 | -74.137 | 26.6 | 35.38 | 715.0 | Caribbean | *S. natans VIII* | MK863947 |
|  | 2018_SN8_126 | 25.897 | -75.618 | 25.7 | 36.71 | 744.0 | S Sargasso | *S. natans VIII* | MK863948 |
|  | 2018_SN8_127 | 24.832 | -74.762 | 26.2 | 36.61 | 698.9 | S Sargasso | *S. natans VIII* | MK863949 |
|  | 2018_SF3_128 |  |  |  |  |  | S Sargasso | *S. fluitans III* | MK863950 |
|  | 2018_SN8_129 |  |  |  |  |  | S Sargasso | *S. natans VIII* | MK863951 |
|  | 2018_SN8_130 | 24.555 | -74.800 | 26.4 | 36.47 | 662.6 | S Sargasso | *S. natans VIII* | MK863952 |
|  | 2018_SN8_131 | 24.368 | -74.905 | 26.3 | 36.52 | 620.6 | S Sargasso | *S. natans VIII* | MK863953 |
|  | 2018_SF3_132 | 25.927 | -73.780 | 25.4 | 36.64 | 579.1 | S Sargasso | *S. fluitans III* | MK863954 |
|  | 2018_SF3_133 | 25.860 | -72.462 | 25.0 | 36.65 | 630.4 | S Sargasso | *S. fluitans III* | MK863955 |
|  | 2018_SF3_134 |  |  |  |  |  | S Sargasso | *S. fluitans III* | MK863956 |
|  | 2018_SF3_138 | 25.938 | -71.832 | 24.6 | 36.75 | 636.8 | S Sargasso | *S. fluitans III* | MK863957 |
|  | 2018_SN8_139 | 25.992 | -70.937 | 24.8 | 36.75 | 625.8 | S Sargasso | *S. natans VIII* | MK863958 |
|  | 2018_SF3_140 | 26.232 | -69.033 | 25.4 | 36.59 | 579.7 | S Sargasso | *S. fluitans III* | MK863959 |
|  | 2018_SF3_141 | 26.488 | -68.088 | 25.9 | 36.66 | 525.6 | S Sargasso | *S. fluitans III* | MK863960 |
|  | 2018_SN8_142 | 26.958 | -65.220 | 25.4 | 36.84 | 534.1 | S Sargasso | *S. natans VIII* | MK863961 |
|  | 2018_SF3_143 | 26.840 | -62.775 | 24.9 | 36.83 | 538.0 | S Sargasso | *S. fluitans III* | MK863962 |
|  | 2018_SF3_144 | 26.290 | -62.775 | 25.0 | 36.86 | 547.5 | S Sargasso | *S. fluitans III* | MK863963 |
|  | 2018_SF3_145 |  |  |  |  |  | S Sargasso | *S. fluitans III* | MK863964 |
|  | 2018_SF3_146 |  |  |  |  |  | S Sargasso | *S. fluitans III* | MK863965 |
|  | 2018_SF3_147 | 28.582 | -63.365 | 23.8 | 36.80 | 546.1 | S Sargasso | *S. fluitans III* | MK863966 |
|  | 2018_SF3_148 | 30.143 | -63.842 | 23.1 | 36.83 | 565.6 | N Sargasso | *S. fluitans III* | MK863967 |
|  | 2018_SF3_149 | 31.387 | -64.155 | 22.6 | 36.27 | 585.4 | N Sargasso | *S. fluitans III* | MK863968 |
|  | 2018_SF3_151 |  |  |  |  |  | N Sargasso | *S. fluitans III* | MK863969 |
|  | 2018_SF3_152 |  |  |  |  |  | N Sargasso | *S. fluitans III* | MK863970 |
|  | 2018_SF3_153 | 32.502 | -64.573 | 23.0 | 36.10 | 683.1 | N Sargasso | *S. fluitans III* | MK863971 |
|  | 2018_SF3_154 |  |  |  |  |  | N Sargasso | *S. fluitans III* | MK863972 |
